# Supplementary material for: Aligning staff schedules, testing, and isolation reduces the risk of COVID-19 outbreaks in carceral and other congregate settings: A simulation study
Source: PLOS Glob Public Health. 2023 Jan 6;3(1):e0001302. doi: 10.1371/journal.pgph.0001302 (PMC10022395; doi:10.1371/journal.pgph.0001302)
Supplement: S1 File — (DOCX) [file pgph.0001302.s001.docx]

Supplementary Information

## Staff schedules


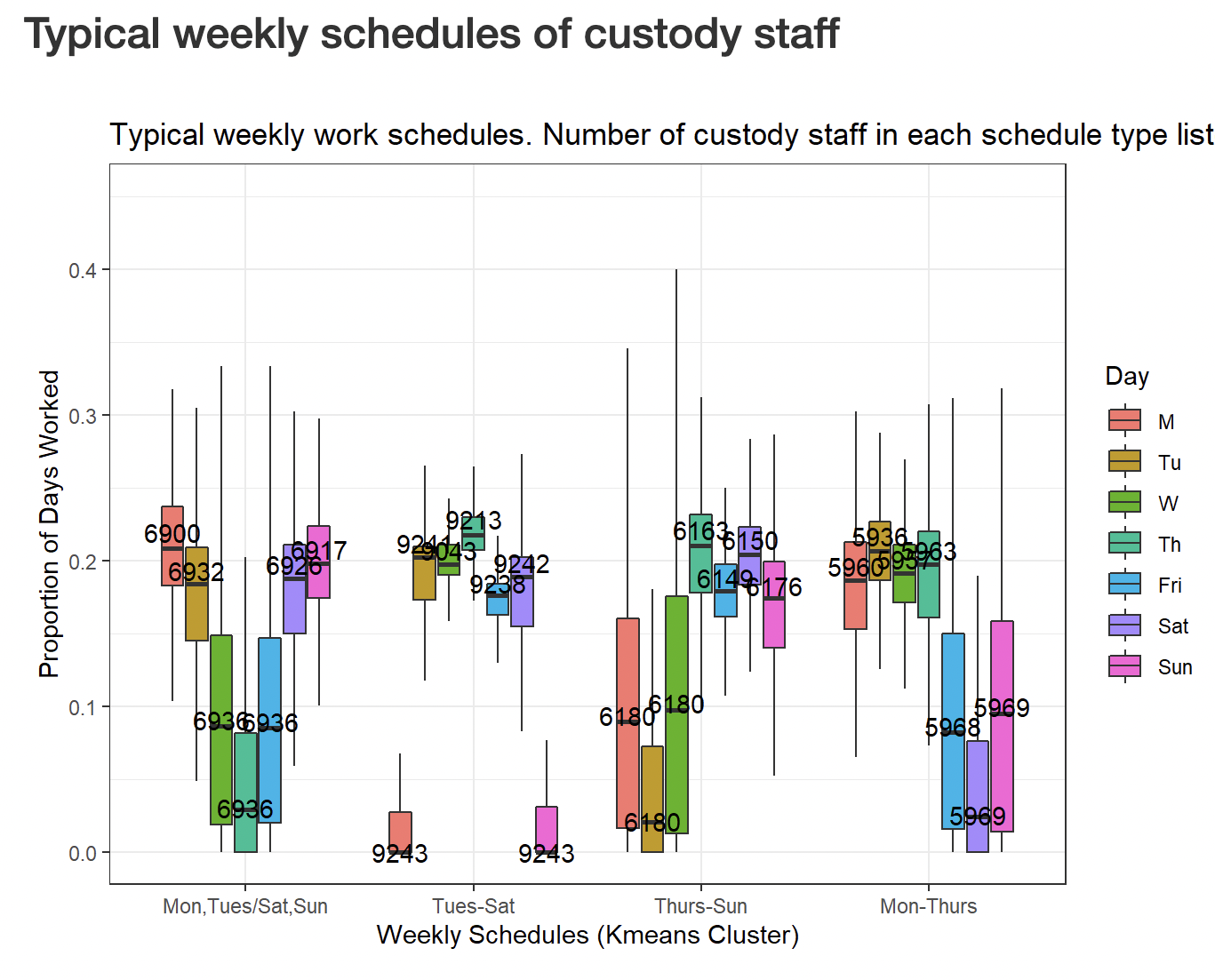


**Fig A: Staff work schedules identified from CDCR operations records via K-means clustering**. Typical schedules are identified along the x axis and boxplots indicate the proportion of shifts worked on each day of the week for the corresponding work schedule. Overlaid numbers indicate the number of staff in CDCR operations records that worked in the identified work schedule. All work schedules were characterized by four or more consecutive workdays (from left to right: Saturday, Sunday, Monday, Tuesday; Tuesday through Saturday; Thursday through Sunday, and Monday through Thursday) with additional shifts commonly occurring prior to or just after these regular 4-day sequences. These structured work schedules were used in model simulations in which systematic testing strategies always occurred on a specified day of the workweek, with testing always on the first day of the workweek leading to the largest reductions in transmission.

## Sensitivity Analyses

Simulations incorporating imperfect test sensitivity and variable isolation delays were conducted. These simulations were meant to compare the tradeoffs between prompt isolation and lower diagnostic sensitivity—as may be expected with the use of rapid antigen tests—to higher sensitivity tests that may result in isolation delays—as may be expected if using NAATs. In addition, simulations relaxing the assumption of no self-isolation due to symptoms were conducted. For these simulations, symptoms were assumed to occur in 80% of SARS-CoV-2 infections and the percent of symptomatic individuals who self-isolate upon symptom onset was varied from 0-100% in 10% increments.

## Sensitivity of tests

Imperfect test sensitivity leading to false negative tests may negatively influence the efficacy of screening testing programs. Because of the importance of limiting delays between testing and isolation of infectious workers, rapid tests with lower sensitivity but quicker results may be more favorable than NAAT tests such as PCR that have very high sensitivity but may take a day or more to determine results. The figure below shows the expected number of cases for different testing frequencies and delays across test sensitivities ranging from 0.8 to 1 (where 20% of tests conducted on infectious individuals would return a false negative with sensitivity of 0.8). All test sensitivity simulations were run with a community prevalence of $1$% and $\mathcal{R=}1.5$.


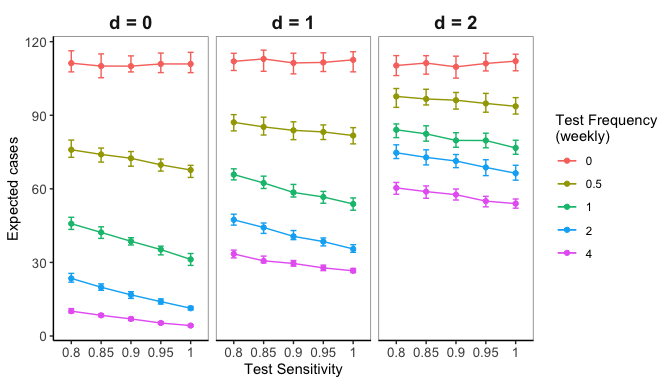


**Fig B: Test sensitivity has less influence than test frequency and delays to isolation on the number of expected infections.**

## Self isolation


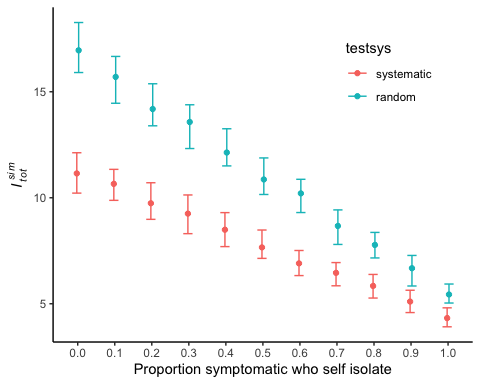


**Fig C: Expected transmissions across probability of self isolating if infection is symptomatic.** Simulations were conducted with weekly testing either systematically on the first day of staff workweeks (red points) or randomly during staff workweeks (blue points), with no delay between testing and isolation of positive cases, and with community prevalence of 0.5% and $\mathcal{R}=1.0$ (corresponding to the middle points of the middle panels in main text figures 3 and 4). 80% of infections were assumed to produce symptomatic cases, with symptom onset occurring at the end of the incubation period at the time of peak infectiousness. More common self-isolation leads to fewer transmission events, and systematic testing strategies have the most benefit when fewer symptomatic cases self-isolate. However, even under perfect self-isolation, systematic testing leads to fewer expected transmission events than random testing. Points indicate the median and error bars represent the interquartile range of expected transmission events ($\mathcal{I}_{tot}^{sim}$) from 100 simulations under each testing strategy.
