# Supplementary material for: Aligning staff schedules, testing, and isolation reduces the risk of COVID-19 outbreaks in carceral and other congregate settings: A simulation study
Source: PLOS Glob Public Health. 2023 Jan 6;3(1):e0001302. doi: 10.1371/journal.pgph.0001302 (PMC10022395; doi:10.1371/journal.pgph.0001302)
Supplement: Attachment — Submitted filename: Response_to_Reviewers.docx [file pgph.0001302.s002.docx]

**Reviewer #1: The authors have described the data inputs, and methods for this simulation study. The authors have also highlighted transparency and ethical aspects through deposition of data, and simulations in a public repository. The manuscript is acceptable in the current form.**

The authors appreciate the reviewer’s kind words and review!

**Reviewer #2: This study examines the relationship between work schedules, testing schedules, and within-facility transmission of SARS-CoV-2 in carceral and other congregate settings using the data from California Department of Corrections and Rehabilitation (CDCR). I would like to congratulate the author(s) to this useful attempt, since limited attempts conducted. Though the manuscript is well-written and the subject addressed in this manuscript is worth of investigation, to allow for a better clarity for the reader and meaningful scientific findings the authors suggested to**

**(a) explain methods section/analysis of data, source, assumptions and validation of data and results further, since methodology is hard to follow;**

We have reviewed the methods again and made edits throughout in an attempt to improve the clarity and logical flow of this section.

**(b) figures given in this manuscript are rather fuzzy and unclear, and give the figures with higher resolution;**

The figures have now been uploaded and reprocessed through the PACE system to ensure they will comply with PLoS guidelines in the final version.

**(c) Title could be modified to indicate the study objective instead of observed results and include study setting/country in the title;**

We appreciate this suggestion and did consider alternative titles such as “Exploring the added benefit of routine SARS-CoV-2 testing by aligning test and staff schedules”, but we are ultimately restricted by character count in the title and feel the current title conveys important aspects of the study including the study design, objective, and results.

**(d) extend further with possible interaction terms;**

The model employed in the study has potential for extension by explicitly incorporating additional interventions. Understanding interactions between the testing strategies explored here and such interventions would be valuable, but we felt beyond the scope f this study. We refer the reviewer to the discussion, lines 349-355 which now states

“In addition, the exclusion of notable COVID-19 prevention strategies (e.g. universal masking, physical distancing, proper ventilation, vaccination) and of additional testing due to symptoms or known contacts is a limitation. However, the sensitivity analysis implementing self-isolation suggests qualitative trends between testing strategies would persist, and only the magnitude of simulated infections would be reduced. Furthermore, these additional interventions would presumably act by reducing $\mathcal{R}$ and are therefore implicitly included in simulations with variable reproduction numbers.”

**(e) discuss the strengths and develop limitations further and especially for each limitation it would be good to say why you believe it is not a major or not affecting the results, including the sources of the data considered, along with implications for further development.**

We appreciate this opportunity to further explain the key limitations and strengths of our analysis. We have made various additions to the limitations and discussion in an attempt to further explain the impact of any limitations on key results.

**Before publication, I would urge the authors to have another good 'go' at the manuscript, post-acceptance, as I am sure they will be able to make further minor improvements in structure and presentation. In conclusion, though the authors have done a good job in addressing the topic, I feel, the manuscript is certainly less disjointed and needs some major improvements on its structure, methodology/analytical framework and presentation of observations etc., before possible consideration of publication for a better clarity and follow the reader.**

The authors appreciate the reviewer’s suggestions and hope that we have adequately responded to them to further improve the manuscript.

**Journal Requirements:**

**1. We have noticed that you have uploaded Supporting Information files, but you have not included a list of legends. Please add a full list of legends for your Supporting Information files after the references list.**

We have added a legend for the included supporting information files after the references list

**2. In the online submission form, you indicated that your data will be submitted to a repository upon acceptance.  We strongly recommend all authors deposit their data before acceptance, as the process can be lengthy and hold up publication timelines. Please note that, though access restrictions are acceptable now, your entire data will need to be made freely accessible if your manuscript is accepted for publication. This policy applies to all data except where public deposition would breach compliance with the protocol approved by your research ethics board. If you are unable to adhere to our open data policy, please kindly revise your statement to explain your reasoning and we will seek the editor's input on an exemption. Please be assured that, once you have provided your new statement, the assessment of your exemption will not hold up the peer review process.**

The only input data for our study were anonymized staff and testing schedules provided by the California Correctional Health Care Services (CCHCS), which we are not at liberty to share publicly. All additional materials such as code, simulated datasets and results are available on github at <https://github.com/cmhoove14/Congregate-Staff-Testing>. We have included the following directions in our Code and Data Availability Section:

**“**Anonymized staff records used to generate figure 2 and inform staff schedules used in simulations can be requested by submitting a Data Request Form for consideration by the California Correctional Health Care Services (CCHCS) Data Advisory Committee (DAC).” **Please review your reference list to ensure that it is complete and correct. If you have cited papers that have been retracted, please include the rationale for doing so in the manuscript text, or remove these references and replace them with relevant current references. Any changes to the reference list should be mentioned in the rebuttal letter that accompanies your revised manuscript. If you need to cite a retracted article, indicate the article’s retracted status in the References list and also include a citation and full reference for the retraction notice.**

We have reviewed the references to ensure that no articles have been retracted. Citation style has also been updated to conform with PLoS guidelines.
